# Supplementary material for: Temporal trends in primary care-recorded self-harm during and beyond the first year of the COVID-19 pandemic: Time series analysis of electronic healthcare records for 2.8 million patients in the Greater Manchester Care Record
Source: eClinicalMedicine. 2021 Nov 1;41:101175. doi: 10.1016/j.eclinm.2021.101175 (PMC8557994; doi:10.1016/j.eclinm.2021.101175)
Supplement: Supplementary file 1 [file mmc1.docx]

**Table S1: Involvement of people with lived experience of self-harm services: GRIPP2 Short-form checklist**
