# Supplementary material for: Temporal trends in primary care-recorded self-harm during and beyond the first year of the COVID-19 pandemic: Time series analysis of electronic healthcare records for 2.8 million patients in the Greater Manchester Care Record
Source: eClinicalMedicine. 2021 Nov 1;41:101175. doi: 10.1016/j.eclinm.2021.101175 (PMC8557994; doi:10.1016/j.eclinm.2021.101175)
Supplement: Supplementary file 2 [file mmc2.docx]

**Table S1: Involvement of people with lived experience of self-harm services: GRIPP2 Short-form checklist**

| Checklist item | Description |
| --- | --- |
| **1: Aim**  Report the aim of the study | To examine monthly trends in GP recorded self-harm episodes before, during and after the first year of the Covid-19 pandemic in Greater Manchester, focussing on the impact of national and regional restrictions and on subgroups of patients. To work with members of an existing panel of people with lived experience of healthcare services for self-harm to interpret and disseminate the findings. |
| **2: Methods**  Provide a clear description of the methods used for PPI in the study | Four panel members were involved in this study, all of whom had prior experience of advising on research studies using electronic health records and some of whom had completed training related to understanding data. Panel members and the lead researcher (SS) met via video call on a regular basis to discuss the interpretation, presentation and dissemination of findings. Panel members reviewed findings based on their experiences of mental illness, primary care, mental health services and the Covid-19 restrictions. |
| **3: Results**  Outcomes—Report the results of PPI in the study, including both positive and negative outcomes | Panel members reviewed the results of the study in terms of the interpretation of findings and the visual presentation of them. The importance of perceived access to primary care in the context of concomitant COVID-19 hospitalisation rates was highlighted. The impacts on different groups of patients, such as existing mental health services users, was discussed. Changes to the graph showing the main results (Figure 1) were proposed by the group, including adding details of schools reopening dates. The importance of an interactive tool, hosted on an accessible website which was separate to the published journal article, was emphasised as part of the planned dissemination of the work. |
| **4: Discussion**  Outcomes—Comment on the extent to which PPI influenced the study overall. Describe positive and negative effects | The involvement of lived experience panel members positively influenced the interpretation, presentation and planned dissemination of the study. There were limitations to the extent that the data extracted from the GMCR was influenced. Not all of the suggestion made by members were possible due to limitations of the data source. |
| **5: Reflections** Critical perspective—Comment critically on the study, reflecting on the things that went well and those that did not, so others can learn from this experience | On reflection, the involvement of lived experience panel members was limited to specific stages of the research, but contributed to important developments of the presentation of results, ensuring accessible forms of dissemination were prioritised. |
